# Biphenyl-2,2′-diyl diacetate

**DOI:** 10.1107/S1600536808035113

**Published:** 2008-11-08

**Authors:** Chen-Hui Hu, Jing Chen, Ji-Cai Quan, Jin-Tang Wang

**Affiliations:** a Department of Applied Chemistry, College of Science, Nanjing University of Technology, Nanjing 210009, People’s Republic of China

## Abstract

In the title compound, C_16_H_14_O_4_, a derivative of 2,2′-biphenol, the benzene rings are oriented at a dihedral angle of 58.32 (3)°.

## Related literature

For bond-length data, see: Allen *et al.* (1987 ▶).
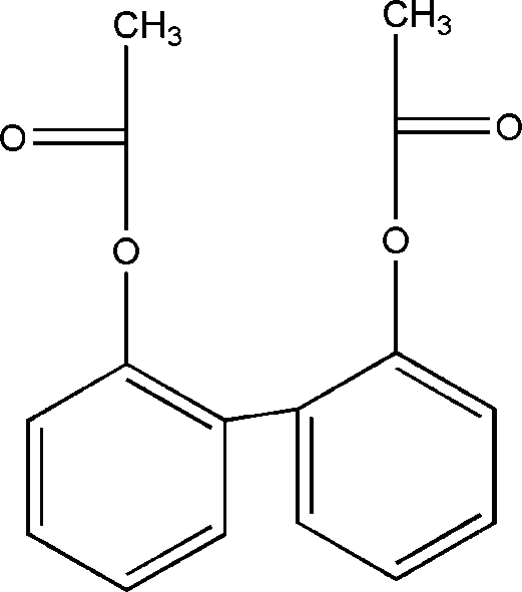


## Experimental

### 

#### Crystal data

C_16_H_14_O_4_
*M* *_r_* = 270.27
Monoclinic, 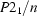

*a* = 8.8380 (18) Å
*b* = 18.204 (4) Å
*c* = 8.9620 (18) Å
β = 108.75 (3)°
*V* = 1365.3 (5) Å^3^
*Z* = 4
Mo *K*α radiation
μ = 0.10 mm^−1^
*T* = 294 (2) K
0.30 × 0.20 × 0.10 mm

#### Data collection

Enraf–Nonius CAD-4 diffractometer
Absorption correction: ψ scan (North *et al.*, 1968 ▶) *T*_min_ = 0.972, *T*_max_ = 0.991
2643 measured reflections
2478 independent reflections
1645 reflections with *I* > 2σ(*I*)
*R*_int_ = 0.026
3 standard reflections frequency: 120 min intensity decay: 1%

#### Refinement

*R*[*F* ^2^ > 2σ(*F* ^2^)] = 0.053
*wR*(*F* ^2^) = 0.156
*S* = 1.00
2478 reflections
184 parameters
H-atom parameters constrained
Δρ_max_ = 0.23 e Å^−3^
Δρ_min_ = −0.27 e Å^−3^

### 

Data collection: *CAD-4 Software* (Enraf–Nonius, 1989 ▶); cell refinement: *CAD-4 Software*; data reduction: *XCAD4* (Harms & Wocadlo, 1995 ▶); program(s) used to solve structure: *SHELXS97* (Sheldrick, 2008 ▶); program(s) used to refine structure: *SHELXL97* (Sheldrick, 2008 ▶); molecular graphics: *SHELXTL* (Sheldrick, 2008 ▶); software used to prepare material for publication: *SHELXTL*.

## Supplementary Material

Crystal structure: contains datablocks global, I. DOI: 10.1107/S1600536808035113/hk2562sup1.cif

Structure factors: contains datablocks x. DOI: 10.1107/S1600536808035113/hk2562Isup2.hkl

Additional supplementary materials:  crystallographic information; 3D view; checkCIF report

## Figures and Tables

**Fig. 1.**
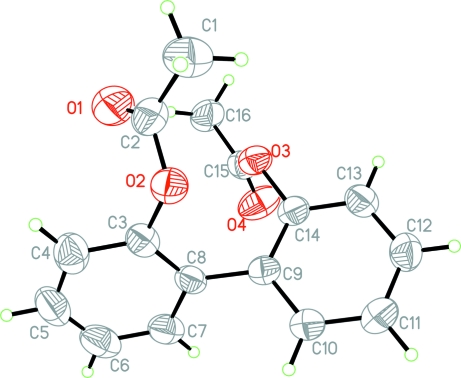
The molecular structure of the title molecule, with the atom-numbering scheme. Displacement ellipsoids drawn at the 30% probability level.

## supplementary crystallographic information

### Comment

Some derivatives of andrographolide are important chemical materials. We report herein the crystal structure of the title compound.

In the title compound (Fig. 1), the bond lengths (Allen *et al.*, 1987) and angles are within normal ranges. Rings A (C3-C8) and B (C9-C14) are, of course, planar and the dihedral angle between them is A/B = 58.32 (3)°.

### Experimental

For the preparation of the title compound, 2,2'-biphenol (10 g) was dissolved in acetic anhydride (50 ml) at room temperature. After the reaction completed, it was extracted with ethyl acetate, washed with saturated salt water and dryed with sodium sulfate. The product was filtrated, and the organic layer was concentrated. Crystals suitable for X-ray analysis were obtained from ethyl acetate (10 ml) at room temperature.

### Refinement

H atoms were positioned geometrically, with C-H = 0.93 and 0.96 Å for aromatic and methyl H, respectively, and constrained to ride on their parent atoms with U_iso_(H) = xU_eq_(C), where x = 1.5 for methyl H and x = 1.2 for aromatic H atoms.

### Crystal data

**Table:**

C_16_H_14_O_4_	*F*_000_ = 568
*M**_r_* = 270.27	*D*_x_ = 1.315 Mg m^−^^3^
Monoclinic, *P*2_1_/*n*	Mo *K*α radiation λ = 0.71073 Å
Hall symbol: -P 2yn	Cell parameters from 25 reflections
*a* = 8.8380 (18) Å	θ = 9–14º
*b* = 18.204 (4) Å	µ = 0.10 mm^−^^1^
*c* = 8.9620 (18) Å	*T* = 294 (2) K
β = 108.75 (3)º	Block, colorless
*V* = 1365.3 (5) Å^3^	0.30 × 0.20 × 0.10 mm
*Z* = 4	

### Data collection

**Table:**

Enraf–Nonius CAD-4 diffractometer	*R*_int_ = 0.026
Radiation source: fine-focus sealed tube	θ_max_ = 25.3º
Monochromator: graphite	θ_min_ = 2.2º
*T* = 294(2) K	*h* = 0→10
ω/2θ scans	*k* = 0→21
Absorption correction: ψ scan(North *et al.*, 1968)	*l* = −10→10
*T*_min_ = 0.972, *T*_max_ = 0.991	3 standard reflections
2643 measured reflections	every 120 min
2478 independent reflections	intensity decay: 1%
1645 reflections with *I* > 2σ(*I*)	

### Refinement

**Table:**

Refinement on *F*^2^	Hydrogen site location: inferred from neighbouring sites
Least-squares matrix: full	H-atom parameters constrained
*R*[*F*^2^ > 2σ(*F*^2^)] = 0.054	*w* = 1/[σ^2^(*F*_o_^2^) + (0.04*P*)^2^ + 2.02*P*] where *P* = (*F*_o_^2^ + 2*F*_c_^2^)/3
*wR*(*F*^2^) = 0.156	(Δ/σ)_max_ < 0.001
*S* = 1.00	Δρ_max_ = 0.23 e Å^−^^3^
2478 reflections	Δρ_min_ = −0.27 e Å^−^^3^
184 parameters	Extinction correction: SHELXL97 (Sheldrick, 2008), Fc^*^=kFc[1+0.001xFc^2^λ^3^/sin(2θ)]^-1/4^
Primary atom site location: structure-invariant direct methods	Extinction coefficient: 0.040 (3)
Secondary atom site location: difference Fourier map	

### Special details

**Table:**

Geometry. All e.s.d.'s (except the e.s.d. in the dihedral angle between two l.s. planes) are estimated using the full covariance matrix. The cell e.s.d.'s are taken into account individually in the estimation of e.s.d.'s in distances, angles and torsion angles; correlations between e.s.d.'s in cell parameters are only used when they are defined by crystal symmetry. An approximate (isotropic) treatment of cell e.s.d.'s is used for estimating e.s.d.'s involving l.s. planes.
Refinement. Refinement of *F*^2^ against ALL reflections. The weighted *R*-factor *wR* and goodness of fit *S* are based on *F*^2^, conventional *R*-factors *R* are based on *F*, with *F* set to zero for negative *F*^2^. The threshold expression of *F*^2^ > σ(*F*^2^) is used only for calculating *R*-factors(gt) *etc*. and is not relevant to the choice of reflections for refinement. *R*-factors based on *F*^2^ are statistically about twice as large as those based on *F*, and *R*- factors based on ALL data will be even larger.

### Fractional atomic coordinates and isotropic or equivalent isotropic displacement parameters (Å^2^)

**Table:**

	*x*	*y*	*z*	*U*_iso_*/*U*_eq_	
O1	0.0543 (3)	0.18629 (18)	0.4765 (3)	0.0742 (8)	
O2	0.0736 (3)	0.22040 (12)	0.7226 (3)	0.0486 (6)	
O3	0.2528 (3)	0.08431 (13)	0.7691 (2)	0.0468 (6)	
O4	0.1896 (4)	−0.03274 (15)	0.7923 (3)	0.0762 (9)	
C1	0.2647 (5)	0.2666 (2)	0.6182 (5)	0.0750 (13)	
H1A	0.2874	0.2719	0.5209	0.113*	
H1B	0.3539	0.2437	0.6952	0.113*	
H1C	0.2463	0.3142	0.6553	0.113*	
C2	0.1200 (5)	0.2203 (2)	0.5920 (5)	0.0544 (9)	
C3	−0.0585 (4)	0.17726 (18)	0.7219 (4)	0.0438 (8)	
C4	−0.2080 (4)	0.1915 (2)	0.6172 (4)	0.0576 (10)	
H4A	−0.2217	0.2269	0.5392	0.069*	
C5	−0.3376 (4)	0.1522 (2)	0.6297 (5)	0.0649 (11)	
H5A	−0.4391	0.1613	0.5596	0.078*	
C6	−0.3171 (5)	0.1001 (2)	0.7448 (5)	0.0656 (11)	
H6A	−0.4047	0.0738	0.7520	0.079*	
C7	−0.1664 (4)	0.0864 (2)	0.8507 (4)	0.0522 (9)	
H7A	−0.1541	0.0511	0.9287	0.063*	
C8	−0.0327 (4)	0.12500 (18)	0.8416 (4)	0.0406 (8)	
C9	0.1255 (4)	0.11569 (17)	0.9635 (4)	0.0395 (7)	
C10	0.1422 (4)	0.12691 (18)	1.1210 (4)	0.0466 (8)	
H10A	0.0525	0.1371	1.1502	0.056*	
C11	0.2908 (4)	0.1231 (2)	1.2353 (4)	0.0546 (9)	
H11A	0.3003	0.1321	1.3401	0.065*	
C12	0.4247 (4)	0.1062 (2)	1.1957 (4)	0.0547 (9)	
H12A	0.5242	0.1039	1.2732	0.066*	
C13	0.4108 (4)	0.09252 (19)	1.0405 (4)	0.0512 (9)	
H13A	0.5005	0.0806	1.0126	0.061*	
C14	0.2620 (4)	0.09669 (17)	0.9267 (4)	0.0418 (8)	
C15	0.2136 (4)	0.0154 (2)	0.7134 (4)	0.0475 (8)	
C16	0.2087 (5)	0.0091 (2)	0.5468 (4)	0.0636 (11)	
H16A	0.1746	−0.0395	0.5088	0.095*	
H16B	0.3134	0.0180	0.5400	0.095*	
H16C	0.1351	0.0445	0.4839	0.095*	

### Atomic displacement parameters (Å^2^)

**Table:**

	*U*^11^	*U*^22^	*U*^33^	*U*^12^	*U*^13^	*U*^23^
O1	0.0705 (19)	0.099 (2)	0.0555 (17)	−0.0022 (17)	0.0229 (14)	−0.0038 (16)
O2	0.0470 (13)	0.0506 (14)	0.0484 (13)	−0.0043 (11)	0.0156 (11)	0.0034 (11)
O3	0.0520 (14)	0.0505 (14)	0.0426 (13)	0.0022 (11)	0.0217 (11)	−0.0004 (11)
O4	0.117 (3)	0.0578 (17)	0.0618 (17)	−0.0175 (17)	0.0403 (17)	−0.0039 (14)
C1	0.069 (3)	0.081 (3)	0.083 (3)	−0.009 (2)	0.034 (2)	0.013 (2)
C2	0.056 (2)	0.056 (2)	0.050 (2)	0.0067 (18)	0.0175 (18)	0.0116 (18)
C3	0.0397 (18)	0.0448 (19)	0.0473 (19)	0.0003 (15)	0.0147 (15)	−0.0042 (15)
C4	0.054 (2)	0.063 (2)	0.051 (2)	0.0104 (19)	0.0100 (18)	−0.0078 (18)
C5	0.040 (2)	0.085 (3)	0.064 (3)	0.009 (2)	0.0099 (18)	−0.016 (2)
C6	0.047 (2)	0.085 (3)	0.071 (3)	−0.012 (2)	0.026 (2)	−0.025 (2)
C7	0.048 (2)	0.060 (2)	0.054 (2)	−0.0084 (17)	0.0233 (17)	−0.0084 (17)
C8	0.0348 (17)	0.0478 (19)	0.0421 (18)	0.0007 (14)	0.0163 (14)	−0.0056 (15)
C9	0.0432 (18)	0.0389 (18)	0.0394 (17)	−0.0009 (14)	0.0172 (14)	0.0023 (14)
C10	0.0465 (19)	0.053 (2)	0.0437 (19)	0.0001 (16)	0.0189 (16)	−0.0018 (15)
C11	0.064 (2)	0.060 (2)	0.0405 (19)	0.0014 (19)	0.0183 (18)	−0.0020 (17)
C12	0.046 (2)	0.063 (2)	0.050 (2)	0.0075 (17)	0.0078 (17)	0.0027 (17)
C13	0.0414 (19)	0.059 (2)	0.055 (2)	0.0038 (16)	0.0175 (16)	0.0047 (17)
C14	0.0463 (19)	0.0433 (18)	0.0383 (17)	0.0032 (15)	0.0168 (15)	0.0020 (14)
C15	0.0423 (19)	0.054 (2)	0.048 (2)	0.0024 (16)	0.0171 (16)	−0.0055 (17)
C16	0.072 (3)	0.077 (3)	0.047 (2)	0.010 (2)	0.0257 (19)	−0.0065 (19)

### Geometric parameters (Å, °)

**Table:**

O1—C2	1.185 (4)	C7—C8	1.400 (4)
O2—C2	1.359 (4)	C7—H7A	0.9300
O2—C3	1.406 (4)	C8—C9	1.482 (4)
O3—C15	1.353 (4)	C9—C10	1.386 (4)
O3—C14	1.406 (4)	C9—C14	1.393 (4)
O4—C15	1.187 (4)	C10—C11	1.383 (5)
C1—C2	1.487 (5)	C10—H10A	0.9300
C1—H1A	0.9600	C11—C12	1.376 (5)
C1—H1B	0.9600	C11—H11A	0.9300
C1—H1C	0.9600	C12—C13	1.379 (5)
C3—C4	1.377 (5)	C12—H12A	0.9300
C3—C8	1.396 (4)	C13—C14	1.384 (5)
C4—C5	1.385 (6)	C13—H13A	0.9300
C4—H4A	0.9300	C15—C16	1.484 (5)
C5—C6	1.370 (6)	C16—H16A	0.9600
C5—H5A	0.9300	C16—H16B	0.9600
C6—C7	1.387 (5)	C16—H16C	0.9600
C6—H6A	0.9300		
			
C2—O2—C3	118.4 (3)	C7—C8—C9	120.9 (3)
C15—O3—C14	116.4 (3)	C10—C9—C14	117.4 (3)
C2—C1—H1A	109.5	C10—C9—C8	120.0 (3)
C2—C1—H1B	109.5	C14—C9—C8	122.5 (3)
H1A—C1—H1B	109.5	C11—C10—C9	120.8 (3)
C2—C1—H1C	109.5	C11—C10—H10A	119.6
H1A—C1—H1C	109.5	C9—C10—H10A	119.6
H1B—C1—H1C	109.5	C12—C11—C10	120.7 (3)
O1—C2—O2	123.7 (4)	C12—C11—H11A	119.7
O1—C2—C1	126.1 (4)	C10—C11—H11A	119.7
O2—C2—C1	110.2 (3)	C11—C12—C13	119.7 (3)
C4—C3—C8	122.4 (3)	C11—C12—H12A	120.1
C4—C3—O2	120.8 (3)	C13—C12—H12A	120.1
C8—C3—O2	116.6 (3)	C12—C13—C14	119.3 (3)
C3—C4—C5	119.0 (4)	C12—C13—H13A	120.4
C3—C4—H4A	120.5	C14—C13—H13A	120.4
C5—C4—H4A	120.5	C13—C14—C9	122.0 (3)
C6—C5—C4	120.4 (4)	C13—C14—O3	117.8 (3)
C6—C5—H5A	119.8	C9—C14—O3	120.2 (3)
C4—C5—H5A	119.8	O4—C15—O3	122.6 (3)
C5—C6—C7	120.3 (4)	O4—C15—C16	126.0 (4)
C5—C6—H6A	119.8	O3—C15—C16	111.4 (3)
C7—C6—H6A	119.8	C15—C16—H16A	109.5
C6—C7—C8	120.8 (4)	C15—C16—H16B	109.5
C6—C7—H7A	119.6	H16A—C16—H16B	109.5
C8—C7—H7A	119.6	C15—C16—H16C	109.5
C3—C8—C7	117.0 (3)	H16A—C16—H16C	109.5
C3—C8—C9	121.8 (3)	H16B—C16—H16C	109.5
			
C3—O2—C2—O1	0.7 (5)	C3—C8—C9—C14	−60.2 (4)
C3—O2—C2—C1	−177.8 (3)	C7—C8—C9—C14	126.0 (3)
C2—O2—C3—C4	−63.2 (4)	C14—C9—C10—C11	3.4 (5)
C2—O2—C3—C8	122.9 (3)	C8—C9—C10—C11	−175.8 (3)
C8—C3—C4—C5	−0.5 (5)	C9—C10—C11—C12	−1.8 (5)
O2—C3—C4—C5	−174.1 (3)	C10—C11—C12—C13	−0.2 (6)
C3—C4—C5—C6	0.1 (6)	C11—C12—C13—C14	0.5 (5)
C4—C5—C6—C7	0.4 (6)	C12—C13—C14—C9	1.2 (5)
C5—C6—C7—C8	−0.4 (6)	C12—C13—C14—O3	178.4 (3)
C4—C3—C8—C7	0.6 (5)	C10—C9—C14—C13	−3.1 (5)
O2—C3—C8—C7	174.3 (3)	C8—C9—C14—C13	176.0 (3)
C4—C3—C8—C9	−173.5 (3)	C10—C9—C14—O3	179.8 (3)
O2—C3—C8—C9	0.3 (4)	C8—C9—C14—O3	−1.1 (5)
C6—C7—C8—C3	−0.1 (5)	C15—O3—C14—C13	96.1 (4)
C6—C7—C8—C9	174.0 (3)	C15—O3—C14—C9	−86.7 (4)
C3—C8—C9—C10	118.9 (3)	C14—O3—C15—O4	−0.8 (5)
C7—C8—C9—C10	−54.9 (4)	C14—O3—C15—C16	−179.6 (3)
